# Surgical Treatment of Infective Endocarditis in Pulmonary Position—15 Years Single Centre Experience

**DOI:** 10.3390/medicina55090608

**Published:** 2019-09-19

**Authors:** Daina Liekiene, Laurynas Bezuska, Palmyra Semeniene, Rasa Cypiene, Virgilijus Lebetkevicius, Virgilijus Tarutis, Jurate Barysiene, Kestutis Rucinskas, Vytautas Sirvydis

**Affiliations:** Department of Cardiovascular Diseases, Faculty of Medicine Institute of Clinical Sciences, Vilnius University, Santariskiu g. 2, Vilnius LT-08661, Lithuania; laurynas.bezuska@gmail.com (L.B.); palmyra.semeniene@santa.lt (P.S.); rasa.cypiene@santa.lt (R.C.); virgilijus.lebetkevicius@santa.lt (V.L.); virgilijus.tarutis@santa.lt (V.T.); jurate.barysiene@santa.lt (J.B.); kestutis.rucinskas@santa.lt (K.R.); vytautas.sirvydis@santa.lt (V.S.)

**Keywords:** infectious endocarditis, pulmonary prosthesis, congenital malformation

## Abstract

*Background and Objectives*: Infective endocarditis in the pulmonary position is a rare disease. Isolated pulmonary valve endocarditis is extremely rare. The aim of our study was to assess patients who were treated surgically for pulmonary endocarditis at our institution from January 2003 to December 2017. *Materials and Methods*: We analyze eight cases of infectious endocarditis in pulmonary position out of 293 patients who were operated for infective endocarditis (2.7%, 8/293). Only two of these eight patients were not related to congenital heart malformation. They were followed for early and late mortality, long-term survival, postoperative morbidity and reoperations. *Results*: Among six patients suffering from congenital heart disease, four patients underwent corrections of pulmonary valve malformation previously, and their infected grafts were replaced by two allografts and two xenografts. The two other patients had replaced their infected pulmonary valves with allografts. Two non-congenital patients with pulmonary valve endocarditis underwent valve replacement with biological prosthesis. All patients survived the early postoperative course. The mean follow-up time was 9.1 (interquartile range (IQR), 5.3–12.6) years. The long-term follow-up included seven patients. One patient (12.5%, 1/8) died more than 4 years after the surgery due to sepsis. Pulmonary endocarditis was the rarest endocarditis treated surgically (*p* < 0.001). *Conclusion*: Surgery for infective endocarditis in the pulmonary position (IEPP) is an effective method of treatment with excellent early outcome and good late results despite a very uncommon pathology and few operations being performed. Surgery performed earlier may make the procedure less radical.

## 1. Introduction

Infective endocarditis (IE) usually damages valves on the left side of the heart, especially the aortic valve. The damage of the heart valves on the right occurs less frequently, occurring in about 5–6% of all endocarditis cases [1]. A lesion in the pulmonary position develops in less than 2% of cases [2,3].

Infective endocarditis in the pulmonary position (IEPP) can be distinguished as either native and non-native tissue endocarditis. It can also be classified as endocarditis in patients with congenital and non-congenital hearts. The latter type is less common, and it is almost exclusively specific to intravenous drug addicts [4,5,6].

The most common infectious agents causing pulmonary endocarditis are *Staphylococci* [1,7,8,9,10]. *Streptococci* is less common [1,11,12]. Seldom the lesion can be due to *Enterococci* [13,14] or *Candida* spp. [2,6]. The treatment of IEPP is always initiated by the administration of antibiotics. Sometimes conservative treatment is successful, and the patients can be cured [4,6]. However, in general, conservative treatment is insufficient and the patients require surgery to remove the infection.

IEPP is a very rare disease and a majority of the articles published include individual cases only. Therefore, we decided to present our surgical experience. A retrospective study of patients with IEPP treated at the Cardiac Surgery Centre of Vilnius University Hospital Santaros Klinikos during the period from 2003 to 2017 is presented in this article. The attention was focused on early and late mortality, long-term survival, postoperative morbidity and reoperations.

## 2. Materials and Methods

Patients who underwent surgical treatment for infective endocarditis in the pulmonary position from January 2003 to December 2017 were identified. All patients were operated on at a single institution at Vilnius University Hospital Santaros Klinikos Centre of Heart and Chest Surgery, Lithuania. The cross-sectional retrospective study was reviewed and approved by the Institutional Review Board of the Hospital (Permission number: 2017-01-09 EK-1, Protocol number DL.1901, approval date 27 May 2019). The requirement for written informed consent was waived due to the retrospective nature of the study. Data were obtained by review of paper and electronic medical records from admission until the last follow-up.

Eight patients were operated on for IEPP during a 15-year-long study period. During this period, 293 patients in total were operated on for IE and only 8 had endocarditis in the pulmonary position. General information on 8 patients with IEPP is given Table 1.

Congenital heart defects (CHD) were diagnosed to 6 (75%) patients and only 2 (25%) patients were not related to congenital heart malformation. The diagnosis was made on the basis of clinical investigation, cardiac imaging, electrocardiography, laboratory data and confirmed by direct observation in the theatre. Blood culture samples were obtained from all patients preoperatively and heart tissue samples were acquired during surgery. Both samples were sent for microbiological analysis and antibiotic sensitivity. Modified Duke criteria were employed for correct and prompt diagnosis. All patients before surgery were treated with antibiotics according to microbiology and sensitivity data. Patients were followed for early and late mortality, long-term survival, postoperative morbidity and reoperations.

### 2.1. Surgical Technique

All operations for IEPP were performed with cardiopulmonary bypass under moderate hypothermia through a median sternotomy. Antegrade tepid blood cardioplegia was used. Types of operations are summarized in Table 2. Two patients with non-congenital IEPP had their damaged valves replaced by biological prostheses; one of these patients concomitantly underwent aortic valve replacement with a biological prosthesis due to IE. Only 2 of 6 congenital patients had native pulmonary valve endocarditis and their infected valves were replaced by allografts concomitantly correcting congenital heart malformations. Replacement of an infected pulmonary conduit was performed for the final 4 patients and one of them also had a replacement of an infected neo-aortic valve with a mechanical prosthesis.

### 2.2. Data Analysis

The statistical software R package (version 3.3.2, R Core Team, 2016) was employed. Descriptive statistics were used to describe characteristics of the study sample. Age was expressed as median and interquartile range, and categorical variables were expressed as frequencies. The chi-square goodness of fit test was used to analyze the differences in localization of endocarditis, heart conditions in patients with pulmonary valve endocarditis and type of congenital heart malformation in patients suffering from congenital heart malformation. Exact *p*-values were calculated wherever appropriate. For multiple comparisons among frequencies of different localizations of endocarditis, Bonferroni correction was applied. All reported *p* values were two-tailed. The level of significance was set at 0.05 [15].

## 3. Results

The mean follow-up time was 9.1 (interquartile range (IQR), 5.3–12.6) years. One patient was lost during follow-up. The age ranged from 3 to 75 years. Median patient age was 22 years (IQR, 15–60.3). The study included 3 (38%) female and 5 (62%) male patients. Distribution of the patients by sex and age are shown in Table 3.

IEPP was the rarest endocarditis treated surgically in our institution (Table 4) (*p* < 0.001). Only 8 patients out of 293 (2.7%) with heart valve IE had IEPP. This lesion was corrected by biological prostheses (25%. 2/8), allografts (50%, 4/8) or xenografts (25%, 2/8). The majority of patients (6 out of 8) with IEPP had concomitant congenital defects, although the statistically significant difference was not reached, probably due to the small sample size (*p* = 0.289).

Four patients (50%, 4/8) had non-native tissue pulmonary endocarditis. Three of them had a Ross procedure performed previously. IEPP in patients with previously implanted pulmonary grafts manifested in a different time after graft implantation. IE developed in less than 3 years for the majority of the patients (75%, 3/4). The period between the primary operation (correction of CHD) and the secondary (conduit replacement) ranged from 18 months to 7 years. The median time of the secondary surgery for IEPP was 3.4 (IQR, 2–3.9) years. Three patients received allografts and 1 patient had a Shelhigh xenograft.

### 3.1. Morbidity

There were no severe postoperative complications in all except 1 patient, who developed renal failure before the surgery and who was on peritoneal dialysis preoperatively and postoperatively. Fever (>38 °C) for a longer time period was present in 4 patients.

The summary of the results of microbiological tests is presented in Table 5. The most common microbiological agents were *Staphylococcus* and *Streptococcus* Spp. Endocarditis for 1 patient was caused by *Enterococcus fecalis*. Microbiological tests failed to reveal microorganisms for 2 patients.

Long term results and condition of the pulmonary grafts are detailed in Table 6. One patient, who had a pulmonary valve replacement with a Shelhigh xenograft, developed degeneration with calcification of the graft. This was causing significant valvular stenosis with a systolic gradient of 64 mm Hg. Nevertheless, the patient felt well in himself, although he may require the conduit replacement in the near future.

### 3.2. Mortality

There was no early mortality. One patient (12.5%, 1/8) died more than 4 years after surgery. This patient was a 15-year-old girl. Her preoperative condition was poor. She had suffered from a febrile status for more than 6 months and hematological disease was suspected. Later on, she developed renal insufficiency and was placed on peritoneal dialysis. Eventually she was admitted to the Pediatric Clinic of Vilnius University and a ventricular septal defect (VSD) complicated by IE damaging the pulmonary valve was diagnosed. Surgery findings included multiple vegetations around both sides of the small perimembranous VSD and a completely destroyed pulmonary valve. The remnants of the pulmonary valve and vegetations were removed. VSD was closed using two U-shaped stitches with Teflon patches. The integrity of the right ventricle and pulmonary trunk was restored using a pulmonary artery allograft. The postoperative course was uneventful. Peritoneal dialysis was continued during the postoperative period and later on, for the rest of her life. At long-term after discharge the patient experienced reoccurring episodes of febrile status and she was treated with antibiotics. However, four and a half years after the surgery the patient died from generalized sepsis.

## 4. Discussion

IEPP is a very uncommon disease. Our institution rate was 2.7% among all patients operated for IE. The incidence in the literature is 2% [1,2,3]. A slightly higher rate might be possibly explained by the fact that we operate on patients with CHD. It is known that the incidence of IEPP is higher for these patients [16].

It is unclear why the right heart valves have a smaller rate of occurrence for IE. It might be the physiological peculiarities of the right side of the heart. Lower oxygen saturation of venous blood in the right chambers may decrease the virulence of aerobic bacteria. However, it is hard to imagine how a lower blood flow decreases the chances of bacteria deployment and adherence to the heart valves. The suggestion could be the initial route of infection is not the most important factor for development of IE as the single contact of bacteria to heart valves is insufficient for deployment and proliferation on endocardium. The constant contact for a longer duration (i.e., bacteremia and sepsis) is possibly needed in order to cause the pathological changes.

The intravenous drug addicts are an exception. They are more susceptible to endocarditis of the right heart valves. A single contaminated intravenous drug infusion contains a very large number of bacteria, which are being deployed on the first valves on route. In accordance with the literature, the infective lesion of a native pulmonary valve occurs almost exclusively in intravenous drug addicts [4,5,6]. Immunosuppression [4,12], long-lasting treatment using central intravenous catheters, permanent foci of infection (dental, intestinal and gonorrhea) are reported as less frequent etiological causes [13]. Contrary to published data both our patients with native pulmonary endocarditis were not drug addicts. They were also not treated with immunosuppressive medications and did not require long-lasting intravenous catheters.

Chahoud [16] in his review paper, noted that pathophysiology of right-sided infective endocarditis is still not very well understood. Among many other factors, the author underlined the importance of the immune complex formation and deposition on the right-sided valves.

As a matter of interest, one of our patients had a combined pulmonary and aortic valves IE. That is a very uncommon pathology [17,18] de Araújo [19] reported the case of aortic and pulmonary IE in a patient who was diagnosed as having VSD as well. It may be suggested that the presence of VSD was a very important factor for the etiology and pathogenesis because turbulent shunt flow could cause damage to the pulmonary valve endothelium. Similar pathogenesis may be suggested for a case of aortic and tricuspid valves IE that developed after the rupture of an aneurysm of the non-coronary sinus of Valsalva into the right atrium [20]. It is very likely that the shunt flow from the aorta affected the tricuspid valve. Birkenkamp’s [17] paper of mitral and pulmonary IE with the presence of VSD may be explained in a similar way. Although our patient did not have a VSD, which makes it even more difficult to explain the pathology. In general, combined endocarditis of the right and left heart is very uncommon [17,18].

In our study, six patients had concomitant congenital heart malformations. VSD or right ventricle infundibular stenosis are considered to be important etiological factors [21,22,23,24] by causing turbulent blood flow and effecting the pulmonary valve. CHD as a direct etiological factor predisposing pulmonary endocarditis could be considered for only two patients. They had no surgery for congenital heart malformations previously. One patient had perimembranous VSD and the other had infundibular and pulmonary stenosis. Pulmonary valve congenital malformation could be the predisposing etiology factor for IE development [25,26]. So, the latter patient had two factors predisposing infection.

The remaining four patients suffered from non-native IEPP and CHD could be considered only as an indirect predisposing factor for IE development. The exact routes of infection were not clear. One of these patients could have had an infection just before the surgery, as preoperatively he had been treated several times with antibiotics for a fever with an unclear etiology. The second patient presumably was infected during the perioperative period. Likely, the other two patients had a new infection as their immediate and long-term postoperative periods were unremarkable. Moreover, grafts are more susceptible to infection in comparison with natural valves [27]. At the long-term period, both allografts and xenografts have a risk for IE at 1% and 1.6% respectively [28,29,30].

Three types of operations could be distinguished for IEPP. The simple one includes removal of vegetations by preserving the valve [24,31]. However, this surgery is the most beneficial at the early stage of infection. Usually, surgeons see the patients after a long treatment with antibiotics, when the pulmonary cusps are damaged. In these cases, the other type of procedure, a pulmonary valve repair, could be advisable. The repair could be performed using auto-pericardium or conserved xeno-pericardium, patching or restoring the cusps of the valve [5]. Unfortunately, long term result is doubtful. The most common way is to replace the affected pulmonary valve with a conduit or biological prosthesis [2,9,10,12,31]. Replacement by mechanical valve prosthesis is being performed very infrequently [22]. All our patients had either a conduit or biological prosthesis for IEPP. The infectious process was outspread with cusps markedly damaged and therefore repair was not feasible. An earlier referral could make surgery less radical.

## 5. Conclusions

Surgery for IEPP is an effective method of treatment with excellent early outcome and good late results despite a very uncommon pathology and seldom operations being performed. Surgery performed earlier may make the surgery less radical.

A retrospective nature and relatively small number of patients are limiting factors for this single institutional study. The change of general improvement in surgical technique and perioperative care has occurred during the study that may alter the long-term results.

## Figures and Tables

**Table 1:** General information of included cases.

No	Diagnosis	Past History	Clinical Presentation	Antibiotic Treatment before Operation	Indications	Operation	Out-Come
1.	Congenital Aortic valve stenosis	Ross operation 2000 y.	2002 y. Fever 39 °C—long term, no response to antibiotics. Sepsis	Oxacillin	Isolated endocarditis of PV allograft	Replacement of the conduit with a new one	Good
2.	CHD—stenosis of PAV and infundibular stenosis of RV	CHD from birth. No surgery or treatment	Sepsis	Oxacillin Gentamycin Cefazolin	Endocarditis of PA	Correction of infundibular stenosis, excision of pulmonary artery valve and restoration of the right ventricle and pulmonary artery integrity using an allograft	Good
3.	Congenital Aortic valve insufficiency	Ross operation 2002 y. 3 month after procedure–fever	Fever, long term, no response to antibiotics, heart failure in progress, AV insufficiency in progress	Amoxicillin Penicillin Vancomycin Unasyn Gentamycin	Endocarditis of AV and PA allograft	Replacement of the graft by an allograft and replacement of aortic valve using mechanical prosthesis (St. Jude)	Good
4.	CHD-VSD	CHD from birth. No surgery or treatment before	Sepsis	Oxacillin Ceftriaxone Cefuroxime Diflucan Meronem Amikacin	Ventricular septal defect and pulmonary artery valve endocarditis	Closure of the defect and replacement of pulmonary artery with an allograft	Good
5.	Congenital Aortic valve insufficiency	Ross procedure 1999 y.	Sepsis 2006	Oxacillin Ampicillin Vancomycin Tienam	Infectious endocarditis of the previously implanted pulmonary artery graft	Replacement of the conduit with a new one	Good
6.	CHD—arterial trunk, VSD	CHD correction with allograft 2005 y.	Sepsis 2007	Vancomycin Ampicillin	Infectious endocarditis of the previously implanted pulmonary artery graft	Replacement of the conduit with a new one	Good
7.	Acute Pulmonary Valve endocarditis	No history	Fever, long term, no response antibiotics, weight loss, heart insufficiency	Unasyn	Isolated pulmonary artery valve endocarditis	Replacement of pulmonary artery valve with a biological prosthesis	Good
8.	Acute Pulmonary Valve endocarditis and Aortic Valve endocarditis	No history	Fever, long term, no response antibiotics. Sepsis	Tazocin Gentamycin	Endocarditis of pulmonary artery and aortic valves	Replacement of aortic and pulmonary artery valves with biological prostheses	Good

**Table 2:** Operations for endocarditis in the pulmonary position.

Diagnosis	Operation	N	Deaths
Isolated pulmonary artery valve endocarditis	Replacement of pulmonary artery valve with a biological prosthesis	1	0
Endocarditis of pulmonary artery and aortic valves	Replacement of aortic and pulmonary artery valves with biological prostheses	1	0
Ventricular septal defect and pulmonary artery valve endocarditis	Closure of the defect and replacement of the pulmonary artery with an allograft	1	0
Infundibular stenosis of the right ventricle, pulmonary artery valvular stenosis and valve endocarditis	Correction of infundibular stenosis, excision of pulmonary artery valve and restoration of the right ventricle and pulmonary artery integrity using an allograft	1	0
Infectious endocarditis of the previously implanted pulmonary artery graft	Replacement of the conduit with a new one	3	0
Endocarditis of pulmonary artery graft and neo-aortic valve (after Ross operation)	Replacement of the graft by an allograft and replacement of aortic valve using a mechanical prosthesis (St. Jude)	1	0
Total		8	0

**Table 3:** Distribution of the patients by sex and age who were operated on for endocarditis in the pulmonary position.

Sex	Age (Years)					Total
	≤10	11–20	21–40	41–60	61–80	
Female		1	1		1	3
Male	1	2		1	1	5
Total	1	3	1	1	2	8

**Table 4:** Frequency distribution of affected heart valves in patients operated for infective endocarditis (IE) from 2003 to 2017.

Affected Valves	Number of Patients	%
Aortic	128	43.7
Mitral	67	22.9
Aortic and mitral	43	14.7
Tricuspid	47	16.0
Pulmonary	6	2.0
Pulmonary and aortic	2	0.7
Total	293	100

Note: Infective endocarditis.

**Table 5:** Distribution of causative microorganisms.

Bacterium	Number of Patients (%)
*Staphylococcus aureus*	2 (25)
*Staphylococcus hominis*	1 (12.5)
*Streptococcus epidermidis*	1 (12.5)
*Streptococcus bovis*	1 (12.5)
*Enterococcus faecalis*	1 (12.5)
Not revealed	2 (25)
Total	8

**Table 6:** Long-term results of surgery for endocarditis in the pulmonary position.

Operation	Date of Surgery	Relapse of Infection	Other Complications	General Condition	Status of the Valve Substitute, Echocardiogram Results
Replacement of pulmonary valve with a biological prosthesis	2014	absent	none	good	valve prosthesis is unremarkable, function is good
Replacement of pulmonary artery valve and aortic valve with biological prostheses	2011	absent	none	satisfactory, mild angina, diabetes	function of the prostheses is good, anatomy is unremarkable
Replacement of infected pulmonary artery allograft with a new conduit	2003	absent	none	good	allograft is unremarkable, function is good, systolic gradient of 30 mm Hg
Right ventricle infundibulectomy. Replacement of pulmonary valve with an allograft	2003				unknown
Replacement of infected pulmonary allograft by a new one and replacement of neo-aortic valve with a mechanical prosthesis	2004	absent	none	good	function of aortic prosthesis and pulmonary allograft is unremarkable
Removal of multiple vegetations of the right ventricle, closure of ventricular septal defect, replacement of pulmonary trunk with an allograft	2004	repeated exacerbations of septic status	continuous preoperative renal insufficiency (on peritoneal dialysis)	poor	no impairment of the valves revealed; late death because of sepsis
Replacement of infected pulmonary artery allograft with a Shelhigh xenograft	2006	absent	none	good	stenosis and calcification of the graft, systolic gradient of 64 mm Hg.

## Abbreviations

IEPP	infective endocarditis in pulmonary position;
IE	infective endocarditis;
CHD	congenital heart disease;
VSD	ventricular septal defect.
